# Prevalence, Risk Factors, and Clinical Management of Disease-Related Malnutrition in Hospitalized Patients: A Descriptive Analysis Using GLIM and SGA Criteria

**DOI:** 10.3390/nu16234099

**Published:** 2024-11-28

**Authors:** Laura Mola Reyes, Rosa M. García-Moreno, Bricia López-Plaza, Samara Palma Milla

**Affiliations:** 1 Clinical Nutrition and Dietetics Unit, Department of Endocrinology and Nutrition, Hospital Universitario La Paz, Paseo de la Castellana, No. 261, 28046 Madrid, Spain; 2 Food, Nutrition and Health Platform, Hospital La Paz Institute for Health Research (IdiPAZ), 28046 Madrid, Spain; bricia.plaza@idipaz.es

**Keywords:** disease-related malnutrition, nutritional assessment, GLIM, SGA, hospitalized patients, risk factors

## Abstract

Objectives: This study aimed to assess the prevalence and risk factors associated with disease-related malnutrition (DRM) in hospitalized patients using the Subjective Global Assessment (SGA) and Global Leadership Initiative on Malnutrition (GLIM) criteria. Additionally, we sought to identify key determinants of moderate and severe malnutrition. Methods: A retrospective analysis was conducted on 1036 adult patients hospitalized in a tertiary care hospital between August 2019 and November 2020. Nutritional status was evaluated using both the SGA and GLIM criteria. Data on demographic characteristics, comorbidities, dietary intake, and gastrointestinal symptoms were collected. Logistic regression models were employed to identify risk factors for DRM, and multivariate analysis was used to determine independent predictors. Results: The prevalence of DRM was 63.3% according to GLIM and 64.8% according to SGA. Moderate malnutrition was observed in 22.6% of patients, while 40.7% were classified as having severe malnutrition, and severe weight loss was noted in 34.5% of the subjects. The key risk factors for DRM included male sex (OR 1.67, *p* < 0.0001), non-oncological gastrointestinal conditions (OR 1.48, *p* = 0.041), infectious diseases (OR 1.66, *p* = 0.007), inadequate ingestion (OR 5.13, *p* < 0.0001), and the presence of gastrointestinal symptoms (OR 3.06, *p* < 0.0001). Individualized diets were found to have a protective effect, while central parenteral nutrition significantly reduced the risk of severe DRM (OR 0.610, *p* = 0.014). In the final adjusted model, sex (*p* < 0.0001), ingestion (*p* < 0.0001), and gastrointestinal symptoms (*p* < 0.0001) emerged as the most significant independent predictors of DRM. Conclusions: The high prevalence of DRM in hospitalized patients emphasizes the importance of routine nutritional screening and personalized interventions. Proactive management of key risk factors such as inadequate intake and gastrointestinal symptoms is crucial to mitigating malnutrition and improving patient outcomes.

## 1. Introduction

Disease-related malnutrition (DRM) is a specific type of malnutrition caused by one or more concomitant diseases, occurring when the severity or persistence of an inflammatory response in an individual leads to the loss of lean body mass or functional impairment [1]. DRM is often present both at the time of admission and at hospital discharge [2].

The prevalence of DRM is notably high among hospitalized patients with complex needs, reaching up to 83% according to the Nutritional Risk Screening 2002 (NRS-2002) and 86% according to the Mini Nutritional Assessment (MNA). Despite its prevalence and significant consequences, DRM is frequently underdiagnosed, and nutritional therapy is often underprescribed [3]. Furthermore, this high prevalence has a profound impact on hospitalization outcomes, such as longer hospital stays, with an average of 6.9 days compared to 4.6 days in well-nourished patients; increased mortality rates during admission, with a 4.4-fold higher risk compared to well-nourished patients; and a greater need for home care or intermediate care facilities upon discharge, with an odds ratio of 2.43 [4,5]. Moreover, malnutrition can become the sole independent predictor of mortality within five months in chronically hospitalized patients with complex needs [6].

The Subjective Global Assessment (SGA) is a widely used tool for diagnosing malnutrition, offering a comprehensive evaluation based on clinical history and physical examination. It has been validated across various populations and is valued for its simplicity and ability to identify patients at nutritional risk. However, the SGA relies on subjective clinical judgment, which may introduce variability in its application [7].

The Global Leadership Initiative on Malnutrition (GLIM) criteria represent a more standardized approach to diagnosing malnutrition, integrating phenotypic and etiologic parameters. These criteria aim to unify global practices and enhance diagnostic consistency by combining objective measures such as weight loss and muscle mass depletion with underlying etiologic factors like inflammation or reduced food intake [8]. GLIM has shown promise in predicting adverse clinical outcomes and aligning with international guidelines, though its implementation may be limited by the challenges involved in reliably measuring muscle mass in some settings.

While SGA has long been recognized as a validated tool for assessing malnutrition, the introduction of the GLIM criteria represents a significant step towards standardizing malnutrition diagnosis globally. However, there remains a gap in the literature regarding how these two approaches compare in clinical practice, particularly in terms of prevalence, diagnostic accuracy, and their implications for patient outcomes.

This makes DRM a significant clinical challenge in the hospital setting. The uncertainty surrounding optimal diagnostic criteria and definitions of malnutrition complicates the identification of patients who could benefit from nutritional interventions [9]. High-quality clinical studies have demonstrated that nutritional therapy can reduce morbidity and other complications associated with malnutrition in certain patients, highlighting the importance of routine malnutrition screening and individualized nutritional interventions as integral parts of clinical care in hospitals worldwide [10,11,12].

In this context, having data from comprehensive samples of hospitalized patients who have undergone nutritional screening is crucial for obtaining accurate evaluations of both the true prevalence of DRM and its associated patterns. Such data provide a solid foundation for the development of targeted interventions. Additionally, a detailed description of the characteristics of patients undergoing nutritional screening is essential for identifying high-risk groups, particularly those with specific diseases, comorbid conditions, or demographic characteristics [13]. This identification is critical for developing prevention and treatment strategies tailored to the needs of specific populations and informing clinical practice guidelines, thereby promoting greater emphasis on nutritional screening and intervention as critical components of hospital care [14].

Furthermore, the variability in diagnostic criteria and nutritional screening tools, as previously noted, is a recognized barrier to the effective identification and management of DRM. In this context, the analysis of extensive patient samples also provides a valuable opportunity to assess the effectiveness of different nutritional screening methods used in clinical practice, thereby contributing to the ongoing debate on best practices for DRM detection [15].

Consequently, this study aimed to conduct a preliminary descriptive analysis of a representative sample of hospitalized patients who underwent nutritional screening, laying the groundwork for future analytical research. In addition to exploring the factors associated with DRM, the study seeks to deepen the understanding of the epidemiology of this condition. This approach not only has the potential to significantly expand the epidemiological database on the prevalence and characteristics of DRM within a specific hospital setting but is also crucial for identifying risk factors and vulnerable populations. Moreover, in subsequent studies, it will allow for evaluation of the effectiveness of various screening and diagnostic tools, providing valuable insights to optimize clinical practice and guide the development of more-effective health policies.

## 2. Material and Methods

### 2.1. Design and Subjects

This single-center nutritional screening study was conducted in a tertiary care hospital, involving adult patients aged 18 years and older who were hospitalized and assessed by the Nutrition and Dietetics Unit between August 2019 and November 2020. The study was conducted in accordance with the Declaration of Helsinki and approved by the Ethics Committee of the participating institution (HULP Code 4430).

### 2.2. Measurement Instruments and Data Collection

Demographic data and clinical assessments were retrieved from each patient’s electronic medical record. Collected variables included sex, age, body weight, height, and BMI, along with additional parameters such as mid-arm and calf circumference, handgrip strength, dietary habits, use of supplementation, albumin levels, sarcopenia, and body composition measured via ultrasound.

GLIM criteria were employed to diagnose malnutrition in adults, adhering to global nutritional guidelines [8]. All components of the GLIM assessment were employed in this study, encompassing phenotypic criteria—comprising three elements—and etiologic criteria. The data for both criteria were obtained according to relevant guidelines [16].

For the first phenotypic component—weight loss percentage—data were collected directly from the patients or, in cases where communication was not possible, from a relative. The specific inquiry was whether the patient had experienced weight loss greater than 5% in the last six months or greater than 10% beyond six months. Height was estimated based on cubit length, and current weight was measured using an electronic scale. BMI was considered low if it was under 20 for patients younger than 70 years and under 22 for those aged 70 years and older. To diagnose malnutrition, patients had to meet at least one phenotypic and one etiologic criterion. The phenotypic metrics used to classify malnutrition severity were Stage 1 (moderate) and Stage 2 (severe).

Additionally, the same nutrition specialist conducted an SGA. The SGA comprises two parts: the first part includes a subjective evaluation of the patient’s nutritional status, including weight loss, dietary intake, gastrointestinal symptoms, and functional capacity. The second part consists of an objective assessment, covering BMI, weight loss, and changes in muscle mass (MM). Following evaluation, each patient was classified based on the specialist’s subjective judgment as A (no malnutrition), B (moderate malnutrition), or C (severe malnutrition). Clinical history, gastrointestinal symptoms, and functional capacity were recorded within the first 24 h of admission. The same specialist also conducted a physical examination to assess the level of muscle atrophy, loss of subcutaneous tissue, and presence of edema. The patient’s functional level was determined by interviewing them or their family.

After an initial screening with the SARC-F tool, sarcopenia was diagnosed based on the criteria defined by the European Working Group on Sarcopenia in Older People (EWGSOP2), which includes low muscle strength (assessed through handgrip dynamometry), low muscle quantity or quality (evaluated using arm muscle circumference), and impaired physical performance (measured via gait speed or other functional tests). Body composition parameters such as fat-free mass, fat mass, and skeletal muscle mass were quantified and standardized according to validated population-specific cutoff points. These measures provided a comprehensive evaluation of nutritional and muscular health, complementing the malnutrition assessments performed in this study.

### 2.3. Data Analysis

The statistical analysis plan was designed to assess the incidence of malnutrition among hospitalized patients and identify potential risk factors associated with this condition. Data were obtained from specific variables recorded for each patient, including but not limited to age, sex, primary diagnosis, and comorbidities. These data were used to identify potential risk factors that might be associated with malnutrition.

For data analysis, an initial descriptive study was conducted to summarize the factors and measurement variables. Frequencies and percentages were calculated for qualitative data, while means, medians, and standard deviations (SD) were used for quantitative variables. Tables and figures were created to facilitate a better understanding of the data. After determining the incidence of malnutrition, a univariate analysis was conducted to evaluate the relationship between each potential risk factor and malnutrition. Factors that showed a statistically significant association were then included in a multivariate logistic regression model. This model allowed for the adjustment of potential confounders and the identification of independent risk factors for malnutrition. To arrive at the final model, the initial models were compared using selection criteria such as the Akaike information criterion (AIC) and the Bayesian information criterion (BIC) to choose the model with the best data fit, which was further validated through goodness-of-fit tests. Multicollinearity among the variables included in the multivariate model was assessed using variance inflation factors (VIFs), ensuring that no significant collinearity was present. The goodness of fit of the final model was further evaluated via the Hosmer–Lemeshow test, confirming its suitability for the dataset. Odds ratios with their corresponding 95% confidence intervals were reported to facilitate interpretation of the relative impact of each predictor variable. Additionally, missing data were handled using complete-case analysis, ensuring the robustness of the statistical findings while minimizing potential biases. An alpha level of 0.05 (CI95%) was considered statistically significant. All analyses were performed using IBM SPSS Statistics for Windows, V25 (IBM Corp., Armonk, NY, USA).

## 3. Results 

### 3.1. Patient Characteristics

**Figure 1:**
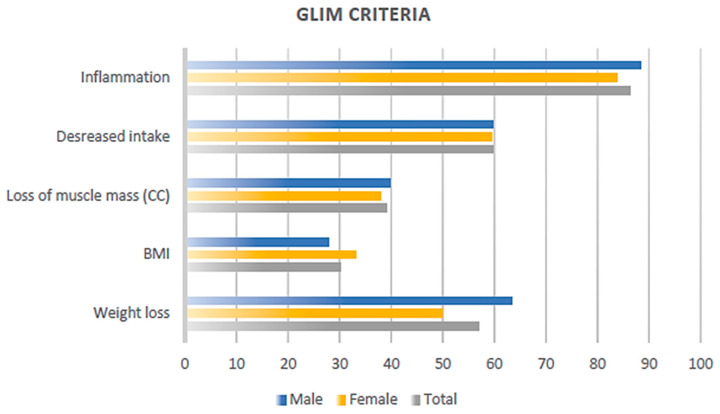
Percentage of patients meeting individual GLIM criteria for malnutrition, stratified by sex. BMI: body mass index; CC: calf circumference.

A total of 1036 hospitalized patients participated in the study, of which 54.5% were male. The mean age was 63.6 (±17.6) years, and nearly half of the subjects (50.6%) were admitted for surgical reasons. Regarding the primary diagnosis, 37.2% of the patients had oncological conditions, 14.5% had non-oncological gastrointestinal conditions, and 15% had infectious diseases. Diabetes was present in 19.2% of the study participants, and the mean BMI was 23.9 (±6.2). Complete patient characteristics and GLIM criteria are detailed in Table 1 and Figure 1.

### 3.2. Prevalence of DRM and Associated Factors

The prevalence of DRM in the study sample was 63.3% according to the GLIM criteria and 64.8% according to the Subjective Global Assessment (SGA). Focusing on the GLIM criteria as the reference for this work, moderate malnutrition was observed in 22.6% of patients, while 40.7% were classified as having severe malnutrition. Additionally, severe weight loss was noted in 34.5% of the subjects (Table 2).

Regarding factors associated with malnutrition, univariate logistic regression revealed a statistically significant difference between males and females, with an odds ratio (OR) of 0.681 (CI95% 0.539 to 0.859), *p* = 0.001, indicating that women have almost half the risk of DRM compared to men (Table 3). Significant differences were also observed for the primary diagnosis variable, with patients that have non-oncological gastrointestinal conditions (OR 1.476, CI95% 1.016 to 2.144, *p* = 0.041) or infectious diseases (OR 1.658, CI95% 1.146 to 2.397, *p* = 0.007) being at higher risk. Patients with gastrointestinal symptoms also showed a significantly higher risk (OR 3.056, CI95% 2.379 to 3.924, *p* < 0.0001), as did those with dysphagia (OR 2.239, CI95% 1.509 to 3.323, *p* < 0.0001).

Regarding the type of diet, taking patients who do not ingest food orally as the reference, statistically significant differences were observed for those on a basal diet (OR 1.451, CI95% 1.050 to 2.005, *p* = 0.024) and those on a therapeutic diet (OR 1.446, CI95% 1.106 to 1.892, *p* = 0.007). However, no significant differences were found for patients on an individualized diet (OR 1.313, CI95% 0.747 to 2.308, *p* = 0.344). This result suggests that adapting the diet to the specific conditions and needs of each patient through individualized diets may be more effective in preventing DRM compared to standard diets.

The use of supplements also showed statistical significance (OR 2.143, CI95% 1.688 to 2.722, *p* < 0.0001), indicating that they are primarily introduced in patients at risk of DRM. Receiving nutritional modules was observed as a protective factor against DRM, with an OR of 0.567 (CI95% 0.395 to 0.814, *p* = 0.002) that indicated statistical significance. Regarding the access route, taking patients without a nasogastric tube or ostomy as the reference, the only variable that was shown to make a difference as a protective factor was the nasogastric tube (OR 0.407, CI95% 0.284 to 0.581, *p* < 0.0001) (Table 3).

### 3.3. Multivariate Regression Analysis

In the multivariate regression analysis, the variables that reached statistical significance and therefore demonstrated independent and differential effects were sex (OR 0.618, CI95% 0.474 to 0.806, *p* < 0.0001), ingestion (OR 5.132, CI95% 3.915 to 6.727, *p* < 0.0001), and gastrointestinal symptoms (OR 2.010, CI95% 1.524 to 2.650, *p* < 0.0001) (Table 4).

### 3.4. Severe vs. Moderate DRM

The multivariate regression analysis conducted to determine independent risk factors for severe DRM, as compared to moderate DRM, identified additional protective factors such as age (OR 0.977, CI95% 0.968 to 0.987, *p* < 0.0001) and central parenteral nutrition (OR 0.610, CI95% 0.411 to 0.906, *p* = 0.014) (Table 5).

## 4. Discussion

The findings from the present study, derived from a representative sample of evaluated subjects, indicate a high prevalence of DRM among hospitalized patients in a third-level hospital, underscoring the urgent need to address this issue systematically and effectively within the hospital setting.

The results are consistent with previous research, highlighting that despite increasing awareness of DRM, current screening and treatment strategies remain insufficient to address the scale of this problem [17]. The high prevalence observed in the present sample not only aligns with earlier studies but also emphasizes the need for more-robust integration of nutritional protocols into routine hospital care [18]. Multidisciplinary teams, for example, have demonstrated improvements in achieving nutritional goals, reducing complications, and optimizing resource use in critically ill patients [19]. Effective integration requires overcoming disciplinary boundaries through structured communication, shared leadership, and mutual understanding of roles [20]. Moreover, implementing standardized protocols for nutritional management has been shown to improve some clinical indicators, such as serum albumin levels, and reduce hospital stays in intensive care settings [21]. Multidisciplinary collaboration is also crucial in oncology, where integrating various professional perspectives improves treatment planning and patient satisfaction [22]. These findings underscore the importance of fostering interdisciplinary teamwork as a cornerstone for addressing the clinical and logistical challenges posed by malnutrition in hospital settings. Without proactive and personalized interventions, DRM will continue to be a critical factor that hinders patient recovery and exacerbates the burden on healthcare systems [23]. It is therefore essential that health policies and clinical guidelines are adapted to reflect the seriousness of this situation, promoting a more comprehensive and evidence-based approach to the detection and management of DRM in hospitals [12].

The present study not only confirmed the high prevalence of DRM but also expanded the understanding of specific risk factors and clinical patterns associated with this condition in a broad and representative sample of hospitalized patients. The analyses revealed that men are at a higher risk of DRM compared to women, highlighting how physiological and metabolic differences influence the ability to maintain nutritional status. Previous studies have indicated that differences in body composition, such as greater muscle mass in men, might make them more susceptible to rapid muscle mass loss during acute illness, contributing to a higher risk of hospital malnutrition [8]. Similarly, chronic inflammation and catabolism, more pronounced in men due to hormonal differences, could accelerate the loss of lean body mass, exacerbating DRM [12]. These findings underline the need for personalized nutritional interventions that consider sex as an important factor in managing DRM in hospital settings [24].

Additionally, the increased risk of DRM found in patients with non-oncological gastrointestinal conditions may be attributed to factors such as nutrient malabsorption, loss of appetite due to pain or discomfort, and the chronic inflammation that often accompanies these diseases. Conditions like inflammatory bowel disease and chronic pancreatitis are strongly associated with nutritional deterioration due to a combination of malabsorption and increased metabolic demands, creating an environment where maintaining adequate nutritional balance is challenging, leading to higher DRM prevalence [25]. Similarly, the increased risk observed in patients with infectious diseases in the present study may be driven by a systemic inflammatory response that increases protein and energy catabolism while simultaneously reducing appetite and food intake [26]. In severe infections such as sepsis, the body’s metabolism is altered, leading to a hyper-catabolic state that significantly increases the patient’s nutritional needs, further exacerbating the risk of DRM [27].

The results of this study also showed that subjects with dysphagia had more than double the risk of developing DRM compared to other patients. This condition, common in patients with neurological, oncological, and other chronic disorders, represents a significant challenge in clinical nutrition as it greatly contributes to inadequate dietary intake and, consequently, to an increased risk of malnutrition [28]. Dysphagia not only affects the quantity of food intake but also the quality of the diet, as patients with this condition may avoid certain textures, limiting the variety and nutritional balance of their diet. This further increases the risk of developing DRM, particularly in those who do not receive adequate management of their condition [29].

From a clinical perspective, these findings emphasize the importance of integrating early and systematic nutritional assessments into routine care: for example, addressing gastrointestinal symptoms with symptom-specific management (e.g., prokinetics or antiemetics) and optimizing the delivery of nutritional support through individualized plans. Moreover, training healthcare teams to recognize and act upon these risk factors—such as developing tailored interventions for at-risk male patients or those with chronic gastrointestinal conditions—could bridge the gap between research and clinical implementation. For instance, prior studies have demonstrated that social and clinical factors like disease severity, polypharmacy, and living alone significantly increase malnutrition risk, reinforcing the need for comprehensive care strategies [30]. Additionally, malnutrition has been associated with increased mortality, hospital stays, and costs, further emphasizing the critical need for effective interventions [31]. Specific predictors such as weight loss and reduced appetite align with findings from other cohorts, suggesting actionable areas for improving care [32].

In this study, notable differences were also observed based on diet type, with patients on basal and therapeutic diets differing from those who do not ingest food orally. This suggests that while basal and therapeutic diets are designed to meet nutritional needs, they may not be sufficient to counteract the impact of disease on the patient’s nutritional status, especially in those with complex conditions or increased metabolic demands [33]. However, patients on individualized diets did not show a significant difference in DRM risk. This finding suggests that individualized diets, tailored specifically to the needs and conditions of each patient, may be more effective in preventing malnutrition compared to standard diets. The personalization of individualized diets allows for greater flexibility in meeting the dietary restrictions and specific nutrient requirements of patients [34]. These findings regarding the type of diet highlight the potential benefits of individualized dietary interventions in hospital settings. While basal and therapeutic diets showed some association with nutritional outcomes, individualized diets did not demonstrate a statistically significant difference in terms of malnutrition in this study. This could reflect the tailored nature of individualized diets, which are designed to address the specific nutritional needs and clinical conditions of each patient, potentially offering better protection against DRM. These results suggest that integrating individualized diets into hospital protocols could improve the effectiveness of nutritional strategies, especially for patients with complex or fluctuating nutritional needs. Further research is needed to explore the impact of individualized dietary approaches on clinical outcomes and evaluate their feasibility and cost effectiveness on a broader scale in diverse hospital environments.

In addition, the results of this study highlighted that the most significant and independent determinants of DRM risk were sex, dietary intake, and gastrointestinal symptoms. As mentioned earlier, inadequate intake of calories and protein is directly responsible for weight loss and muscle mass depletion, leading to DRM. This finding emphasizes the critical importance of continuous nutritional intake monitoring in hospitalized patients and the need for proactive nutritional interventions, such as oral supplements or enteral nutrition, to prevent malnutrition and improve clinical outcomes [35]. Similarly, gastrointestinal symptoms not only directly affect patients’ ability to ingest and absorb nutrients but are also associated with increased catabolism and protein loss, as discussed earlier, exacerbating the risk of malnutrition [36]. The confirmation by the current study of these independent risk factors for DRM further highlights the need to guide the implementation of more-personalized preventive and therapeutic strategies in hospital settings, with early intervention aimed at reducing the prevalence of DRM.

Building on these observations, future research should focus on developing and validating more-precise and accessible tools for the early detection of DRM across diverse hospital settings. Additionally, exploring the effectiveness of personalized nutritional interventions in specific populations, such as patients with chronic gastrointestinal conditions or severe infections, would be valuable to optimize clinical outcomes. Longitudinal studies could also provide deeper insights into the temporal dynamics of DRM, including the impact of comorbidities, nutritional interventions, and disease progression on nutritional status. These research directions would not only address current methodological limitations but also guide the implementation of more-effective clinical protocols and health policies.

On the other hand, one critical aspect to address is the relative strengths and limitations of the GLIM criteria compared to the SGA tool. While both methods identified similar prevalence rates of malnutrition, evidence from the literature highlights differences in diagnostic performance and clinical utility. Studies report moderate agreement between GLIM and SGA, with kappa values ranging from 0.22 to 0.80 depending on the population and context, reflecting variability in their overlap [37]. GLIM has shown higher specificity but lower sensitivity, potentially missing some malnourished patients but being more accurate in confirming the diagnosis [38]. Furthermore, GLIM has demonstrated stronger predictive capacity for long-term outcomes, such as mortality [39]. Practical challenges remain with GLIM, particularly in the assessment of muscle mass and inter-rater variability when applying its criteria. Standardizing its application and further exploring its diagnostic concordance with SGA would help clarify their complementary roles in clinical practice [40].

However, the main limitation of this study arises from its use of a prevalent sample, which may introduce biases that affect the interpretation of certain findings. By design, prevalent samples capture patients at a specific point in time, potentially failing to reflect the dynamic nature of disease progression and recovery or the fluctuating risk of DRM during hospitalization. This approach may overrepresent patients with stable or chronic conditions while underestimating the impact of acute changes in nutritional status. Additionally, certain variables, such as comorbidities and length of hospitalization, may not have been fully accounted for in the analysis, influencing the observed associations with DRM. For example, unmeasured variations in comorbidity severity or the timing of nutritional interventions could act as confounders, distorting the true relationship between DRM and clinical outcomes. To address these limitations, future studies should adopt longitudinal designs or incident sampling methods to better capture the temporal dynamics of malnutrition and its predictors, providing a more comprehensive understanding of its clinical trajectory and impact.

A particularly noteworthy limitation is the unexpected finding that older age appeared to act as a protective factor against severe DRM. This result, while seemingly paradoxical, is likely due to survivor bias. In a cross-sectional sample of hospitalized patients, older individuals who remain in the hospital may represent a subgroup with enhanced resilience or more-favorable health profiles, having survived earlier critical stages of illness or malnutrition. Consequently, these older patients may be less likely to exhibit severe malnutrition at the time of assessment. Conversely, younger patients, especially those with acute conditions, may experience a more rapid nutritional decline, explaining their higher rates of severe DRM. Thus, while age appears to be protective in this context, it may reflect a bias in the sample rather than an intrinsic protective effect of aging. Future longitudinal studies are needed to address this limitation and provide a more accurate understanding of the relationship between age and DRM severity.

## 5. Conclusions

In conclusion, the results of this study reinforce the understanding of DRM as a prevalent and multifactorial issue in hospital settings. The identification of risk factors such as sex, inadequate dietary intake, and gastrointestinal symptoms underscores the need for a more personalized and proactive approach to nutritional assessment and management in hospitalized patients. This study therefore underscores the urgency of implementing evidence-based nutritional strategies aimed at reducing DRM and consequently improving clinical outcomes and optimizing hospital resources. 

Expanding on these considerations, translating these findings into clinical practice requires careful consideration of both their potential benefits and the logistical hurdles they may entail. While integrating nutritional risk factors such as gastrointestinal symptoms and sex into routine assessments is feasible with existing tools, ensuring consistent application across diverse hospital settings may require additional training and resource allocation. Moreover, the adoption of interventions like central parenteral nutrition, though effective, could be limited by cost, availability, and the need for specialized expertise. These practical barriers highlight the importance of developing scalable strategies that balance resource constraints with the need for individualized care.

## Figures and Tables

**Table 1:** Demographic and clinical characteristics of the study population.

Variable	Total	Female	Male
Age (mean, sd)	63.6 (17.6)	64 (18.28)	63.3 (16.96)
Sex (n, %)	1036	471 (45.5)	565 (54.5)
Type of care (n,%)			
Medical patients	512 (49.4)	238 (50.5)	274 (48.5)
Surgical patients	524 (50.6)	233 (49.5)	291 (51.5)
Primary diagnosis (n,%)			
Gastrointestinal cancer	142 (13.7)	60 (12.7)	82 (14.5)
Gastrointestinal (no cancer)	150 (14.5)	76 (16.1)	74 (13.1)
Infectious	155 (15)	64 (13.6)	91 (16.1)
Neurological	104 (10)	56 (11.9)	48 (8.5)
Oncology	244 (23.5)	105 (22.3)	139 (24.6)
Others	241 (23.3)	141 (28.1)	100 (19.0)
Diabetes (n%)	199 (19.2)	78 (39.2)	121 (60.8)
Weight (n,%)			
Normal weight	424 (42.8)	226 (50.2)	198 (36.7)
Moderate	224 (22.6)	82 (18.2)	142 (26.3)
Severe	342 (34.5)	142 (31.6)	200 (37.0)
BMI (mean, sd)	23.9 (6.2)	23.85 (6.68)	23.86 (5.68)
Gastrointestinal symptoms (n,%)	458 (45.3)	215 (46.9)	243 (53.1)
Dysphagia (n,%)	133 (13.1)	49 (36.8)	84 (63.2)
Diet (n,%)			
Basal	216 (21.0)	91 (19.4)	125 (22.2)
Individualized	52 (5.1)	29 (6.2)	23 (4.1)
Therapeutics	378 (36.7)	171 (36.5)	207 (36.8)
Nothing by mouth	384 (37.3)	177 (37.8)	207 (36.8)
Supplements (n,%)	488 (47.2)	211 (43.2)	277 (56.8)

BMI: body mass index; sd: standard deviation; n: number of subjects; %: percentage.

**Table 2:** Nutritional status assessment by SGA and GLIM, stratified by sex.

Variable	Total	Female	Male
SGA (n,%)			
Good nutritional status	364 (35.2)	189 (40.1)	175 (31.1)
Moderate malnutrition	331 (32.0)	147 (31.2)	184 (32.7)
Severe malnutrition	339 (32.8)	135 (28.7)	204 (36.2)
GLIM (n,%)			
Good nutritional status	379 (36.6)	194 (41.3)	185 (32.8)
Moderate malnutrition	234 (22.6)	95 (20.2)	139 (24.6)
Severe malnutrition	421 (40.7)	181 (38.5)	240 (42.6)

SGA: Subjective Global Assessment; GLIM: Global Leadership Initiative on Malnutrition; n: number of subjects; %: percentage.

**Table 3:** Nutritional access routes and supplementation.

Variable	B	Wald	OR	OR (CI95%)		*p*-Value
Supplements	0.762	39.057	2.143	1.688	2.722	<0.0001
Nutritional modules	−0.568	9.463	0.567	0.395	0.814	0.002
Access Route						
No feeding tube or ostomy		27.368				<0.0001
Gastrostomy	0.373	1.731	1.452	0.833	2.530	0.188
Nasogastric tube	−0.900	24.348	0.407	0.284	0.581	<0.0001
Nasojejunal tube	20.574	0.0	8.62 × 10^8^	0	-	0.999
Jejunostomy	20.574	0.0	8.62 × 10^8^	0	-	0.999
Others	20.574	0.0	8.62 × 10^8^	0	-	1.000

OR: odds ratio; CI: confidence interval.

**Table 4:** Multivariate logistic regression for DRM based on the GLIM criteria and the adjusted final model.

Variable	B	Wald	Adjusted OR	Adjusted OR(CI95%)		*p*-Value
Sex	−0.480	12.608	0.618	0.474	0.806	<0.0001
Ingestion	1.635	140.256	5.132	3.915	6.727	<0.0001
GS	0.698	24.485	2.010	1.524	2.650	<0.0001
Constant	−0.401	11.690	0.669			0.001

GS: gastrointestinal symptoms; OR: odds ratio; CI: confidence interval.

**Table 5:** Multivariate logistic regression for severe vs moderate DRM and the adjusted final model.

Variable	B	Wald	Adjusted OR	Adjusted OR (CI95%)		*p*-Value
Age	−0.23	21.933	0.977	0.968	0.987	<0.0001
Intake	0.355	3.824	1.426	0.999	2.036	0.051
GS	0.364	4.976	1.439	1.045	1.982	0.026
PN						0.023
PN-Central	−0.495	6.001	0.610	0.411	0.906	0.014
PN-Peripheral	−0.425	2.751	0.654	0.395	1.080	0.097
Constant	1.758	23.359	5.803			<0.0001

GS: gastrointestinal symptoms; PN: parenteral nutrition; OR: odds ratio; CI: confidence interval.

## Data Availability

The data presented in this study are available from the corresponding author upon request.
